# Prévalence de l'hypertension artérielle dans la population des meuniers de la ville de Lubumbashi, République Démocratique du Congo

**DOI:** 10.11604/pamj.2015.22.152.6677

**Published:** 2015-10-16

**Authors:** Léon Kabamba Ngombe, Karen Cowgill, Ben Bondo Monga, Benjamin Kabyla Ilunga, Wembonyama Okitotsho Stanis, Oscar Luboya Numbi

**Affiliations:** 1 Unité de Toxicologie et Environnement, Ecole de Santé Publique, Faculté de Médecine, Université de Kamina, République Démocratique du Congo; 2 Fulbright Scholar, Ecole de santé Publique, Université de Lubumbashi, République Démocratique du Congo; 3 Ecole de Santé Publique, Faculté de Médecine, Université de Lubumbashi, République Démocratique du Congo; 4 Université de Lubumbashi, Faculté de Médecine, Département de Pédiatrie, République Démocratique du Congo; 5 Hôpitaux Universitaires de Strasbourg, France

**Keywords:** Hypertension artérielle, meuniers, bruits, environnement du milieu de travail, hypertension, millers, noise, workplace

## Abstract

**Introduction:**

L'objectif était de comparer la prévalence de l'hypertension artérielle chez les meuniers dans la ville de Lubumbashi qui sont exposés d'une manière permanente aux bruits des machines de transformation des céréales à la prévalence de l'hypertension artérielle chez un groupe des gardiens.

**Méthodes:**

Quic'est une étude descriptive transversale qui a concerné 286 meuniers et 115 agents d'une entreprise de gardiennage. Apres un consentement éclairé, un auto questionnaire a été administré aux enquêtés et des mesures de poids, taille, et tension artérielle ont été prises.

**Résultats:**

La prévalence de l'HTA chez les meuniers était 49.3% et celle des gardes était 20.9%, pour un ratio de prévalence de 2.4. significativement élevée par rapport aux contrôles (49.3% vs 20.9%) et le ratio de prévalence était de 2.4. Les facteurs de risque tels que: l’âge, l'indice de masse corporelle, l'ancienneté, la durée de travail ont été significativement élevés chez les meuniers par rapport aux gardiens.

**Conclusion:**

Nous avons trouvé une prévalence élevée de l'HTA chez les meuniers. Nos résultats suggèrent que cette prévalence est probablement due à l'environnement du milieu de travail des meuniers (bruits, vibration des machines) et ce dernier constitue un facteur de risque de l'hypertension artérielle.

## Introduction

Selon le BIT, la meunerie est l'ensemble des opérations de traitement et de broyage des céréales qui conduisent à la production de farines destinées à être consommées par l'homme et par les animaux. L'environnent du milieu de travail des meuniers indique que ces derniers sont exposés aux bruits, aux vibrations, aux poussières, au stress et sont susceptible de développer plusieurs pathologies telles que l'asthme, l'HTA, les rhinites. En effet, l'hypertension artérielle (HTA) est la pathologie et le facteur de risque cardiovasculaire le plus important. Actuellement, on estime qu'environ un milliard de personnes dans le monde souffre d’ HTA et ce chiffre devrait encore augmenter avec le vieillissement de la population [1]. Elle est également un facteur de risque cardio-vasculaire majeur dans la survenue d'accidents vasculaires cérébraux, d'insuffisance cardiaque, d'insuffisance rénale et de maladies coronaires qui représentent les principales causes de décès dans le monde [2–4]. Elle touche toutes les races, toutes les ethnies et toutes les couches socioprofessionnelles [5]. De nos jours, on estime qu'environ un milliard de personnes dans le monde souffre d’ HTA et ce chiffre devrait encore augmenter avec le vieillissement de la population [6]. Elle est inégalement répartie selon les continents et selon les pays. C'est ainsi que 15% de la population française, 20% de la population américaine, 18% de la population chinoise sont concernées par l'HTA [7]. Dans la région africaine, 20 millions de personnes seraient affectées [8]. Les accidents vasculaires cérébraux figurent parmi les premières complications rapidement fatales de l'HTA [9]. L'HTA n'est plus une maladie de l'Occident car elle touche également les pays en développement [10, 11]. En RD Congo, bien que la prévalence de l'HTA ne soit pas clairement connue et documentée, Philippe B. Katchunga la situaient à 41% en milieu urbain et à 38% en milieu rural au sein de la population Congolaise dans la province du Sud Kivu en 2011 [12], prouvant que cette affection est un réel problème de santé publique. Il y a plus de 20 ans que M'Buyamba-Kabangu avaient trouvé une prévalence de l'HTA de 30% en milieu rural par rapport à 16,7% en milieu urbain [13, 14] avec l’âge avancé comme déterminant majeur pour le milieu rural. A Lubumbashi, nous n'avons pas encore des données concernant la prévalence de l'hypertension artérielle. De plus, cette pathologie dont les facteurs de risque habituels sont l’âge, la sédentarité, le stress et l'obésité se développe avec acuité dans nos milieux en développement. Cependant, il s'avère important de connaitre la situation de l'HTA en milieu professionnel à Lubumbashi, en particulier chez les meuniers dans la ville de Lubumbashi. Certains auteurs révèlent des fortes prévalences d'HTA chez les travailleurs en Afrique et dans les pays industrialisés [15–17]. L'objectif de cette étude est de comparer la prévalence de l'HTA chez les meuniers avec les gardes, et d'identifier les facteurs favorisant le développement de cette affection chez les meuniers dans la ville de Lubumbashi qui sont exposés d'une manière permanente aux bruits des machines de transformation.

## Méthodes

### Type d’étude, cadre d’étude et population

Il s'agit d'une étude descriptive transversale dans la population des meuniers de chaque commune de la ville de Lubumbashi au Katanga. Seuls les meuniers ayant plus de 6 mois d'ancienneté ont participé à l'enquête. Le groupe contrôle est composé de 118 gardes d'une société de gardiennage de la place ayant plus de 6 mois d'ancienneté avec le même niveau socio-économique que les meuniers. Il est important de savoir que les gardes ont été engages sans un examen médical au préalable et sans qualificatif (l'embauche des gardes est très sélective) d'ou ils sont comparable aux meuniers. La ville de Lubumbashi est la deuxième ville de la République démocratique du Congo la plus peuplée après Kinshasa, sa population avoisinerait deux millions d'habitants d'après les dernières estimations. C'est la capitale économique de la RDC ; elle est également la capitale de la province du Katanga. Elle est désignée comme la capitale cuprifère à cause de la production du cuivre et fait partie du Copper Belt Africain. Cette étude a été menée en 2014 sur une période de 3 mois, allant du 1^er^ Mai au 31 Juillet 2014 dans les 7 communes de la ville de Lubumbashi. Dans notre enquête un questionnaire général portant sur les caractéristiques sociodémographiques et professionnelles des meuniers et des contrôles a été utilisé. Le poids a été mesuré au moyen d'une balance calibrée et vérifiée, la taille a été mesurée avec une toise. Un indice de masse corporelle a été calculé comme rapport du poids(en kilogrammes) au carré de la taille (en mètres) [18]. Les sujets ayant un IMC > 30 Kg/m^2^ ont été considérés comme obèses. L'enregistrement de la pression artérielle au moyen d'un appareil électronique (OMRON Hem8402) était effectué au bras droit soutenu à hauteur du cœur, le sujet s’étant reposé pendant au moins 10 minutes en position assise. Les tensions artérielles ont été prises par le même observateur et répétées plus de trois fois chez les sujets en position assise tout en respectant l'intervalle de 10 minutes. La moyenne de trois mesures consécutives a servi pour l'analyse. Nous avons considéré comme hypertendu tout sujet dont la pression artérielle systolique était supérieure ou égale à 140 mm Hg et/ou la pression diastolique supérieure ou égale à 90 mm Hg [19]. La confidentialité et l'anonymat ont été garantis aux personnes qui ont répondue aux questions. Les enquêteurs ont bénéficié d'une formation au préalable et l'enquête a été réalisée par entretien direct entre les enquêteurs et les personnes incluses dans l’étude. Le consentement éclairé des sujets a été obtenu de ces derniers.

### Echantillonnage

La taille de l’échantillon était fonction du nombre des meuniers que nous avons trouvé sur le lieu de service lors de notre descente. Ainsi on a procédé à un échantillonnage exhaustif pour arriver à avoir tous les meuniers de la ville de Lubumbashi commune par commune. Ainsi, notre échantillon était constitué de 115 contrôles et de 286 meuniers sélectionnés exhaustivement. La répartition des 286 meuniers selon les différents postes de travail se présentait de la manière suivante : maïs, 44 sujets (15%), manioc et soja, 2 sujets (1%), manioc 16 sujets (5%), mais-manioc et soja, 45 sujets (16%), mais et manioc, 180 sujets (63%).

### Description brève des conditions de travail des meuniers

**Figure 1:**
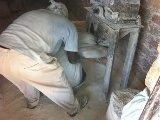
Description brève des conditions de travail des meuniers

L'environnement du milieu de travail des meuniers indique que ces derniers sont exposés aux bruits et aux vibrations d'une manière permanente générés par les moteurs et les équipements de transformation des céréales, aux poussières de farines de différentes céréales lors de la transformation (broyage) à cause de la vétusté des équipements de transformation; à des fortes chaleurs et à des températures élevées générées par le manque d'espace ou par manque d'un système de ventilation et d'aération adéquat. Le manque des matériels de protection individuelle et collective caractérise également ce milieu. Toutes ces mauvaises conditions de travail créent un stress pouvant avoir de répercussion sur la santé des meuniers (Figure 1).

### Statistiques

Excel 2010, EPI info7 ont été utilisés pour faire les analyses. Les données ont été présentées sous forme de fréquences ou de moyenne ± écart-type selon le cas. Le Chi carré avec ou sans correction de Yates a été utilisé pour la comparaison des proportions d'HTA de l’échantillon. Le seuil de signification choisi correspondait à une valeur de α de 0.05.

## Résultats

**Figure 2:**
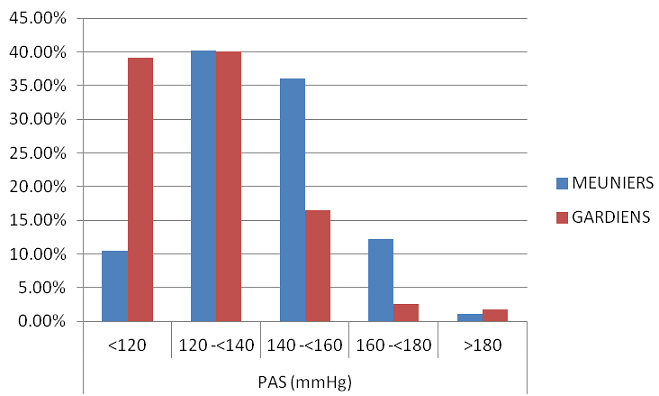
Répartition de la pression artérielle systolique

**Figure 3:**
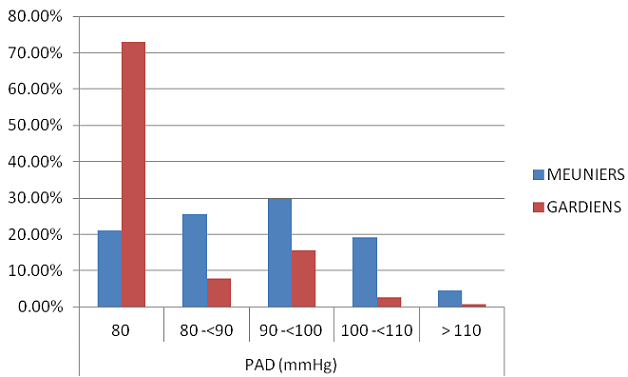
Répartition de la pression artérielle diastolique

**Figure 4:**
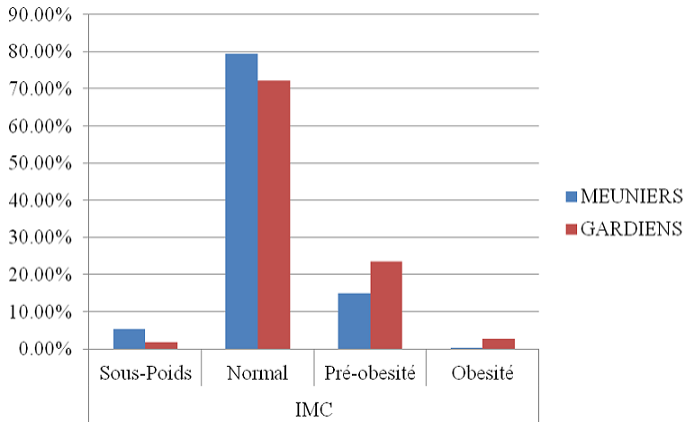
Répartition de l'indice de masse corporelle

Notre population est constituée de 286 meuniers et 115 gardiens. L’âge des meuniers était de 27,42±9 contre 28.57±8 chez les gardiens, l'ancienneté des meuniers était de 4.8±5.5 ans contre 5±4.5 ans chez les gardiens. La durée de travail était de 12.09±1.7 heures/jour chez les meuniers contre 14.53±6.2 heures/jour chez les gardiens. Quant à l’éducation, 12.94% des meuniers étaient diplômé contre 45.22% des gardiens. Concernant l’état civil, 44.41% des meuniers étaient marié contre 76.52% des gardiens. Chez les meuniers, 43.36% prenaient de l'alcool contre 13.91% des gardiens. Pour le tabac, on a trouvé que 31.47% des meuniers étaient des fumeurs contre 14.78 des gardiens (Tableau 1). Le ratio de prévalence pour l'exposition primaire c.-à-d. l'occupation (ou la profession) était de 2.4, ce qui montre que les meuniers avaient une prévalence de l'HTA 2 fois supérieur à la prévalence des gardiens. On note que 36% des meuniers ont une pression artérielle systolique comprise entre 140 et <160 mm Hg contre 16.5% chez les gardiens (Figure 2). Concernant la pression artérielle diastolique, 29.72% des meuniers sont dans la tranche 90-<100 mm Hg par rapport à 15.65% chez les gardiens (Figure 3). Cependant, il existe d'autres facteurs importants qui diffèrent entre les deux groupes et qui pourraient expliquer cette différence. En ce qui concerne l’âge, la prévalence de l'HTA était de 3 fois plus élevée chez les meuniers que chez les gardiens dans la tranche d’âge <30 ans, tandis qu'elle est encore de 2 fois élevée chez les meuniers que chez les gardiens dans la tranche d’âge ≥ 30 ans. Pour l'IMC, la prévalence de l'HTA était de 2.6 fois supérieure chez les meuniers que chez les gardiens dans la tranche d'IMC<25 et dans la tranche d'IMC ≥ 25, la prévalence de l'HTA était 2 fois plus élevée chez les gardiens que chez les meuniers. On a noté également que 0.35% des meuniers était obèse contre 2.61% des gardiens (Figure 4). Selon l'ancienneté, les meuniers ont présenté dans la tranche d'ancienneté <5ans une prévalence de l'HTA 2.7 fois supérieure par rapport aux gardiens et dans la tranche d'ancienneté ≥ 5ans, la prévalence est de 1.8 fois élevée chez les meuniers que chez les gardiens. Concernant la durée de travail, on a noté une prévalence de l'HTA 3 fois plus élevée chez les meuniers que chez les gardiens dans la tranche <12 heures/jour et dans la tranche ≥ 12 heures/j, la prévalence de l'HTA est de 1.9 fois élevée chez les gardiens que chez les meuniers. Quant au tabac, nous avons également noté une prévalence de l'HTA 1.7 fois plus élevée chez les meuniers prenant le tabac que chez les gardiens ; et qu'il était important de savoir que le facteur tabagisme n’était pas significatif pour l'HTA. La prévalence de l'HTA chez les meuniers prenant l'alcool était 2 fois plus élevée par rapport aux gardiens. Ce chiffre n’était pas significatif car le facteur alcoolisme n'influence pas à lui seul l'HTA (Tableau 2).

**Tableau 1:** Répartition anthropométrique et sociodémographique

Caracteristiques Anthropometrique/Socio-Demographiques	Meuniers	Gardiens
	N (%)	N (%)
**Age**	27.4±9	28.6±8
Anciennete (Ans)	4.8±5.5	5±4.5
Duree De Travail (Heures)	12.1±1.7	14.5±6.2
Imc	22.1±2.4	23.3±3.6
Pas	140.3±15.9	126.1±17.4
Pad	90±12.1	73.9±12.2
**Education**		
Primaire	41(14.3)	6(5.2)
Secondaire	208(72.7)	57(49.6)
Diplome	37(12.9)	52(45.2)
**Etat Civil**		
Marie	127(44.4)	88(76.5)
Celibataire	157(59.9)	26(22.6)
Divorce	2(0.7)	0
Veuf	0	1(0.9)
**Alcool**		
Oui	124(43.4)	16(13.9)
Non	162(56.6)	99(86.1)
**Tabac**		
Oui	90(31.5)	17(14.8)
Non	196(68.5)	98(85.2)

**Tableau 2:** Facteurs de risque de l'HTA chez les meuniers

Facteurs Recherches	Meuniers		Gardiens		Pr	Ic95%
	Tension artérielle		Tension artérielle			
	Normale N(%)	Elevée N(%)	Normale N(%)	Elevée N(%)		
Profession	145 (50.7)	141 (49.3)	91 (79.1)	24 (20.9)	2.4	[1.6-3.4]
**Age**						
<30 ans	95 (33.2)	91 (31.8)	64 (55.7)	12 (10.4)	3.1	[1.8-5.3]
≥30 ans	40 (14)	60 (21)	27 (23.5)	12 (10.4)	2	[1.2-3.2]
**IMC**						
<25	137 (47.9)	126 (44.1)	76 (66.1)	17 (14.8)	2.6	[1.7-4.1]
≥25	15 (5.2)	8 (2.8)	7 (6.1)	15 (13)	2.1	[1-4.1]
**Ancienneté**						
<5 ans	108 (37.8)	108 (37.8)	62 (53.9)	14 (12.2)	2.7	[1.7-4.4]
≥5 ans	37 (12.9)	33 (11.5)	29 (25.2)	10 (8.7)	1.8	[1-3.3]
**Durée du travail**						
<12 H/jour	132 (46.2)	129 (45.1)	51 (44.3)	10 (8.7)	3	[1.7-5.4]
≥12 H/jour	13 (4.5)	12 (4.2)	40 (34.8)	14 (12.2)	1.9	[1-3.4]
**Tabac**						
Non	108 (37.8)	88 (30.8)	80 (69.5)	18 (15.7)	2.4	[1.6-3.8]
Oui	37 (12.9)	53 (18.5)	11 (9.6)	6 (5.2)	1.7	[0.8-3.2]
**Alcool**						
Non	70 (24.5)	92 (32.2)	79 (68.7)	20 (17.4)	2.8	[1.9-4.3]
Oui	55 (19.2)	69 (24.1)	12 (10.4)	4 (3.5)	2	[0.9-5.3]

## Discussion

Notre travail est une étude descriptive transversale portant sur 286 meuniers avec une moyenne d’âge de 27.42±9 et 115 gardiens avec une moyenne d’âge de 28.57±8. Les meuniers de la ville de Lubumbashi ont été sélectionnés d'une manière exhaustive malgré que cet échantillon ne soit pas représentatif de toute la population de la province ou du pays. Cependant, les limites de notre étude ont été le nombre restreint des meuniers, la discrimination imposée par les mauvaises conditions de travail en faveur des hommes d'où la majorité masculine dans ce domaine, et donc on ne peut s'attendre à un effet sexe. La modalité de prises de la tension artérielle en une seule visite, même si elle a été répétée plus de 3 fois chez les meuniers et les gardiens fait partie aussi des limites de notre travail. La prévalence de l'HTA chez les meuniers dans notre enquête était de 49.3% par rapport à 20.9% chez les gardiens. Cette différence de prévalence est significative entre les deux groupes. Ainsi, la prévalence de l'HTA chez les meuniers dépasse largement celle estimée par KOFFI chez les travailleurs du secteur public du Port Autonome d'Abidjan [20] et celle de Ta-Yauan Chang chez les travailleurs exposés aux bruits en Taiwan [21]. Cette prévalence est également plus élevée que celle trouvée dans la population du Sud Kivu par Katchunga [12], par M'Buyamba-Kabangu dans la population Congolaise [13, 14], par Comoe dans les populations ouest africaines [22]. Certaines enquêtes menées en milieu de travail montrent que la prévalence d'HTA dépendait de la profession et des conditions de travail [21, 23]. Plusieurs auteurs ont confirmé cela : Valles M. [24] Chez les travailleurs du milieu hospitalier, Naceur [25] chez le personnel du commerce, et Idahosa P.E. [26] Chez les policiers et les fonctionnaires au Nigeria. Dans la littérature, il existe à l'heure actuelle des auteurs qui ont révélé un lien entre l'exposition aux bruits d'un environnement professionnel de façon chronique ou permanente et l'hypertension artérielle [21, 27–31]. En effet, le stress, les facteurs psychosociaux [32] et l'inactivité physique [33] sont incriminés dans la survenue de l'hypertension artérielle. La forte prévalence de l'HTA chez les meuniers de la ville de Lubumbashi pourrait être due premièrement aux conditions de travail néfastes (bruits et vibrations permanents engendrés par les machines de broyage associés au manque de matériels de protection) qui ont été constatées dans ce milieu de travail. Ainsi, l'idéale serait de mesurer les différents niveaux sonores et des bruits auxquels les meuniers sont exposés.

Il est connu que le risque de survenue de l'HTA devient fréquent et augmente massivement avec l’âge. C'est ainsi qu'il représente chez les meuniers et les gardiens 31.8% et 10.4% dans la tranche d’âge <30 ans; 21% et 10.4 % dans la tranche d’âge ≥30 ans. Ceci illustre que nos résultats sont différents avec les données de la littérature qui associent souvent l'HTA aux sujets âgés [14, 34]. L'obésité représente un facteur de risque majeur de l'HTA. Cependant, notre étude a trouvé: 2.8% des meuniers et 13% des gardiens étaient des obèses hypertendus. Cette observation n'est pas conforme aux enquêtes des autres auteurs menées à ailleurs [20, 35–37]. Selon Chang, l'ancienneté au poste de travail et le degré d'intensité forte aux bruits d'une manière chronique auxquels sont exposés les travailleurs sont associés à une élévation de la pression artérielle, et constituent un facteur de risque élevé de l'HTA [21]. Dans notre étude, nous avons trouvé une prévalence de l'HTA élevée chez les meuniers ayant une ancienneté inferieure ou supérieure à 5ans avec une durée de travail <12h par rapport aux gardiens. Cependant, nos résultats vont dans le même sens avec ceux trouvés par d'autres chercheurs [21, 38]. Le tabac est un facteur de risque retrouvé chez 18.5% des meuniers hypertendus par rapport à 5.2% des gardiens dans notre enquête. Cette prévalence chez les exposés n'est pas différente de celle trouvée par Nouhoum à Bamako [39]. Mais dans notre étude, le tabac est un facteur de risque non significatif car il ne peut pas à lui seul influencer l'HTA chez les meuniers. Néanmoins, il est connu de la littérature que l'influence du tabagisme sur la pression artérielle est à prouver avec exactitude. Par ailleurs, Baer et Radichevich ont constaté une élévation moyenne de la PAS de 11 mm Hg et de la PAD de 9 mm Hg après une cigarette dans un groupe d'hypertendu. Ceci montre l'influence du tabac sur la pression artérielle, et pourrait être investigué chez les meuniers. Dans notre enquête la prévalence de l'HTA chez les alcooliques meuniers est de 24.1% contre 3.5% chez les gardiens. Notre prévalence de l'alcoolisme est supérieure à celle trouvée par d'autres auteurs [39, 40] mais inferieure par rapport à celle de l’étude de Drabo [41]. En ce qui concerne d'autres auteurs [42–44] la corrélation alcool, pression artérielle est une relation continue sans seuil. Par contre, certains auteurs révèlent un seuil au-dessous duquel une faible consommation n’élèverait pas la pression artérielle et tendrait même peut être à la faire diminuer [45, 46]. En effet, une fréquence élevée de l'HTA chez les sujets ayant une forte consommation d'alcool confirme l'association entre l'alcool et la pression artérielle. Il est connu de nos jours que l'alcoolisme peut expliquer à lui 10 à 30% des HTA chez certains sujets selon d'autres auteurs [42, 43, 46–48]. Ceci mérite d’être appondis chez les meuniers de la ville de Lubumbashi car le facteur de risque alcool n’était pas significatif. Tous ces facteurs bien que suffisamment documentés sont mal connus dans nos pays en développements, et surtout dans nos différents milieux professionnels en particulier chez les meuniers où l'HTA gagne du terrain à cause des mauvaises conditions de travail de ces derniers et par le manque d'un système d’éducation sanitaire et de la médecine du travail dans le milieu des meuniers.

## Conclusion

Au terme de notre étude nous pouvons conclure que les meuniers ont une prévalence de l'HTA deux fois supérieure à celle des gardiens, et que les facteurs de risque tels que le tabac, l'alcool n’étaient pas significatifs. Nos résultants révèlent que la profession des meuniers dans les pays sous-développés pourrait être un métier dangereux et constituerait un sérieux problème de santé publique. Par ailleurs, notre étude suggère que l'exposition aux bruits générés par les machines des broyages est associée à un risque d'HTA chez les meuniers. Il s'avère nécessaire de mener une étude de cohorte dans la population des meuniers, et d'instaurer impérativement la médecine du travail dans ce milieu afin de développer les mesures préventives tout assurant une sensibilisation permanente pour réduire les risques professionnels.
